# Longitudinal relations between child emotional difficulties and parent-child closeness: a stability and malleability analysis using the STARTS model

**DOI:** 10.1186/s13034-024-00777-1

**Published:** 2024-07-15

**Authors:** Ioannis G. Katsantonis, Jennifer E. Symonds, Ros McLellan

**Affiliations:** 1 Faculty of Education, University of Cambridge, 184 Hills Road, CB2 8PQ Cambridge, UK; 2 Institute of Education, Faculty of Education and Society, University College London, London, UK

**Keywords:** Emotional mental health, Parent-child closeness, Parent-child relationship, STARTS model, Reciprocal relations, Longitudinal cohort study, Bayesian structural equation modelling, Growing Up in Ireland

## Abstract

**Background:**

Past empirical evidence on the longitudinal relations between emotional mental health symptoms and parent-child close relationships has produced mixed and inconclusive results. Some studies suggest a unidirectional relation, whereas other studies point toward a bidirectional association. Additionally, most of the past research has been carried out with adolescent samples, rather than children. Hence, this study aimed to estimate the longitudinal relations between children’s trait emotional difficulties and trait parent-child closeness, accounting for the time-invariant and time-varying state components of each factor.

**Methods:**

Participants were 7,507 children (ages 3 years, 5 years, 7 years, and 9 years) from the Growing Up in Ireland cohort. Α bivariate stable trait, autoregressive trait, and state (STARTS) model was estimated using Bayesian structural equation modelling.

**Results:**

The STARTS model revealed that children’s emotional difficulties and parent-child closeness were relatively stable across time, and these overarching traits were strongly negatively correlated. Children’s earlier trait emotional difficulties predicted later trait parent-child closeness and vice versa between 3 years and 5 years, and between 5 years and 7 years, but these effects disappeared between 7 years and 9 years. At all pairs of time points, state emotional difficulties and state parent-child closeness were weakly negatively correlated.

**Conclusions:**

Overall, the results suggest that early and middle childhood are critical stages for improving parent-child relationships and reducing children’s emotional difficulties. Developing close parent-child relationships in childhood appears to be a key factor in reducing children’s subsequent emotional difficulties. Children who face greater than usual emotional difficulties tend to be more withdrawn and less receptive to close parent-child relationships and this could serve as an important screening indicator.

**Supplementary Information:**

The online version contains supplementary material available at 10.1186/s13034-024-00777-1.

## Background

Epidemiological evidence has highlighted a spike in children’s emotional difficulties in middle childhood around 9 years old [1]. Emotional mental health difficulties span a range of internal, covert symptoms, and overcontrolled behaviours that are critical risk factors for social and academic maladjustment [2]. Hence, we need to examine the factors that can prevent the development of increased emotional difficulties. One such critical factor is close parent-child relationships, which can protect against the potential development of worsening emotional difficulties in childhood and adolescence [3, 4]. Yet, according to the transactional model of child development [5], the parent-child relationship may also change because of the child’s emotional difficulties. Previous evidence studying the long-term relations between parent-child relationships and child emotional difficulties has produced inconclusive evidence regarding the bidirectional association between parent-child closeness and child emotional difficulties. Moreover, past evidence has focused more on the adolescent period [6–9], rather than across early and middle childhood.

Hence, the current study examines the longitudinal dynamics between parent-child closeness and child emotional mental health across early and middle childhood. The study provides an original account of this issue by considering both inter-individual and intra-individual differences. Furthermore, robustness checks are conducted by estimating a conditional model to ensure that the findings are robust to the inclusion of known covariates.

### Longitudinal associations between parent-child closeness and child emotional health

Parent-child interactions in general can have a decisive influence on children’s mental health development [10]. Attachment theory clearly outlines the positive manifold influence of nurturing parent-child relationships on children’s later-life cognitive and socio-emotional development [11]. A key component of attachment is parent-child relationships. Parent-child relationships can be characterised either by conflict (e.g., struggle, anger, etc.) or by closeness/ connectedness [12, 13]. Parent-child closeness is defined as sharing affectionate, warm, and responsive relationships with the child [14, 15]. Although parent-child conflict has been recognised as a threat to children’s mental health [4], less is known about the impact of parent-child closeness [12].

Parent-child closeness can be a strong protective factor against increased child mental health difficulties [16]. Yet, studies report mixed results regarding the directional nature of the longitudinal association between parent-child close relationships and child emotional symptoms. For instance, some scholars found a rather weak predictive effect from parental warmth and closeness to adolescent emotional symptoms [6, 17]. Meanwhile, other studies have shown a reciprocal association between parent-child closeness and emotional functioning [16]. Of note, a study that disentangled state and trait variance components, but not measurement error, had also indicated a weak negative reciprocal relation between parental warmth and emotional difficulties, namely, depression [18]. In contrast, other studies reported no predictive relation over time [8, 9]. Another state-trait study on parental warmth and adolescent emotional difficulties (i.e., depression) revealed mixed results, with depression predicting subsequent decrements in warmth at specific time points and warmth negatively predicting subsequent depression also at specific time points [7]. In short, the above evidence appears to be rather inclusive regarding the bidirectionality between parent-child closeness and child emotional difficulties in addition to being more concentrated around the adolescent years.

Given the above, the current study addresses these mixed findings by investigating the bidirectional relationships between parent-child closeness and child emotional symptoms using the more advanced STARTS model, which separates stable traits, autoregressive traits, states, and measurement error [19]. By focusing on early to middle childhood from a trait-state perspective, we aim to provide a clearer understanding of these dynamic associations in a rather under-researched developmental period.

### A state-trait perspective on parent-child closeness and child emotional health: the STARTS approach

A critical limitation of past studies on the longitudinal association of parent-child closeness and child emotional symptoms is that they typically ignore the fact that human cognitive processes, emotions, and behaviours are characterised by both stability and change over time [20]. Thus, we cannot measure future parent-child closeness and child emotional symptoms in a situational vacuum as the State-Trait theory (LST) would suggest [21]. Research on adult [22, 23] and child [4, 24] psychopathology has illustrated that mental health displays both trait-like time-invariant and time-varying (state-like) features. Although there is scarce evidence for parent-child closeness (an exception is Tran et al., 2023), it is highly plausible that closeness will be characterised by both change and stability. Hence, we need to disentangle these different sources of variance when estimating the longitudinal relations between emotional mental health and parent-child closeness.

**Fig. 1:**
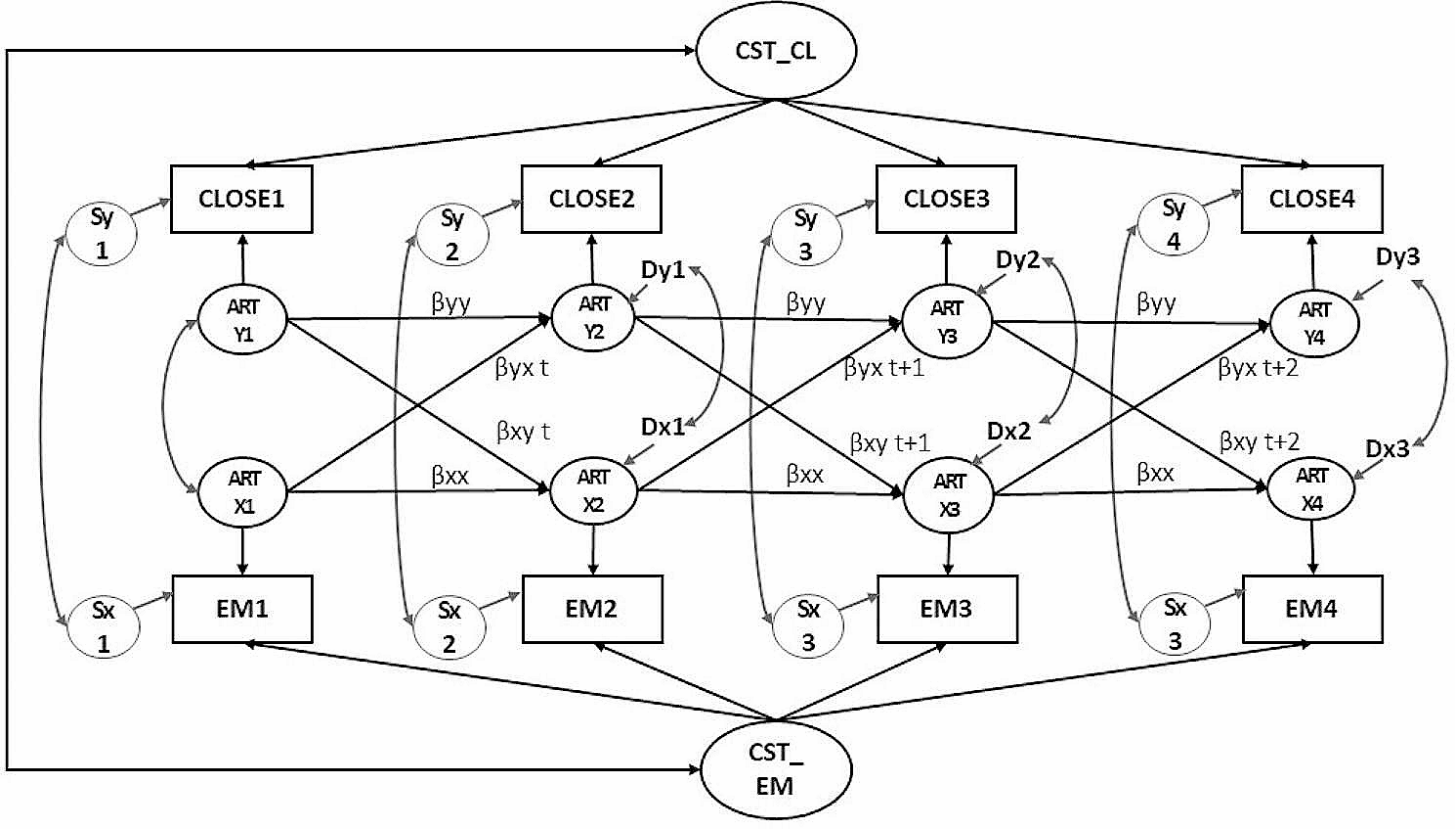
Bivariate stable trait, autoregressive trait, and state (STARTS) model.  CST_EM: Completely stable trait emotional symptoms; CST_CL: Completely stable trait closeness; EMt: Observable emotional symptoms at time t; CLOSEt: Observable closeness at time t; ART Xt: Autoregressive trait of emotional symptoms at time t; ART Yt: Autoregressive trait of closeness at time t; Dyt/Dxt: Disturbance at time t; Sxt/Syt: State at time t; β: Linear regression coefficient

To achieve the above aim, the Stable Trait, Autoregressive Trait, and State (STARTS) model [19], previously known as the Trait-State-Error model [25], is an ideal analytic model that teases apart sources of variance attributable to different time-invariant and -varying components. The STARTS model estimates three components, namely stable traits, autoregressive traits, and states [26]. The stable traits capture long-lasting completely time-invariant variance [19]. Stable traits represent inter-individual differences and dispositions [20]. The autoregressive trait component is a partially enduring component that is still subject to predictable change through a lag-1 autoregressive process [26]. The model also estimates a completely occasion-specific state component that is partially capturing measurement error [19]. The multivariate extension of this model to estimate the longitudinal bivariate association of child emotional symptoms and parent-child closeness is depicted in Fig. 1. The STARTS model clearly separates the measurement error of the manifest variables from the developmental processes taking place within individuals [27, 28]. By separating measurement error, we can get more reliable and accurate estimates [29] and we can estimate within-person longitudinal relations [28]. Hence, the STARTS model is a very appropriate analytic choice for the current study.

However, the STARTS model cannot account for every possible omitted variable. Therefore, it is important to conduct a robustness check by statistically controlling for other covariates [30]. Hence, we control for standard demographic factors that are known to influence children’s mental health in addition to children’s and parents’ general health levels [4, 10].

### The present study

In brief, the current study contributes to the existing literature in three important ways. First, the study partitions the variance in child emotional difficulties and parent-child closeness into completely stable traits, autoregressive traits, and states. Second, having distinguished between stability and malleability in the two factors in childhood, the directional relation between child emotional mental health difficulties/symptoms and parent-child closeness was examined from age 3 years to 9 years. This is an important contribution to extant literature because the majority of previous studies have investigated this longitudinal association mostly in the adolescent years [6, 8, 9] and, therefore, we do not know much about the within-child and within-parent dynamics from early childhood to middle childhood. Third, previous empirical evidence comes from studies that have not clearly separated measurement error from the cross-lagged effects when estimating the bidirectional relations between parent-child closeness and child emotional difficulties. To that end, the application of the bivariate STARTS model [19] in the Bayesian framework can perhaps provide more realistic estimates of the association of interest over time [27]. Overall, the two research questions are:

RQ1: How stable and malleable are child emotional difficulties and parent-child closeness?

RQ2: What is the nature of the association between child emotional difficulties and parent-child closeness in childhood?

Five working hypotheses were formulated. First, drawing upon the developmental cascades approach to psychopathology [31], we expect bidirectional effects between parent-child closeness and child emotional difficulties (H1). Second, we assumed a negative influence of parent-child closeness on children’s emotional difficulties (H2). Given that some studies showed a null effect [8, 9], it might be possible that parent-child closeness and child emotional difficulties might be weakly or not at all associated (H3). Alternatively, we hypothesise that as children face greater emotional difficulties than usual, parents might face difficulties in approaching and understanding their children’s emotions and children might become more withdrawn. Hence, greater than usual emotional difficulties might predict less than usual parent-child closeness (H4). Finally, we formulate another hypothesis such as children with greater than usual emotional difficulties might seek more close relationships with their parents as a coping mechanism (H5). Some support for this hypothesis comes from resilience research whereby sensitive caregiving, close relationships and social support can act as protective factors [32].

## Method

### Participants

The data were sourced from the longitudinal Growing Up in Ireland cohort study [33–35]. Four waves of the infant cohort were utilised, collected at ages 3-years (*N* = 9,793), 5-years (*N* = 9,001), 7-years (*N* = 5,344), and 9-years (*N* = 8,032) years old. After longitudinal matching, the analytic sample size was 7,507 dyads (parent-child). 49.67% of the children were girls and 50.33% were boys. Children were mostly in households of managerial and technical social class (34.23%), followed by professional workers (19.12%). Most primary caregivers identified as Irish (84.25%) and were mothers (> 95%).

### Measures

#### Emotional mental health symptoms

The emotional symptoms scale of the Strengths and Difficulties Questionnaire (SDQ) [36, 37] was used at 3 years, 5 years, 7 years, and 9 years to assess the children for emotional symptoms. The primary caregivers were asked to rate their children’s mental health symptoms. The scale comprises five items, each scored using a three-point scale ranging from 0 “not true” to 2 “certainly true”. The SDQ emotional symptoms scale is a standardised measure and is predictive of some of the most common mood disorders [38]. The scale has good reliability coefficients across waves, ranging between 0.69 and 0.70. Sample items include “[the Child] has many worries” and “[the Child] has many fears, is easily scared”. The composite summed score ranged from 1 to 10. Higher scores indicate greater emotional mental health difficulties.

#### Parent-child relationship-closeness

The Pianta Parent-Child Closeness scale [13, 39] was administered to the primary caregivers of the children at ages 3 years, 5 years, 7 years, and 9 years. This scale is made up of seven items and indexes warm, affectionate, and responsive parent-child relationships [39]. Sample items include “I share an affectionate, warm relationship with [the Child]”, “[the Child] openly shares feelings and experiences” and “it is easy to tune in to child’s feelings”. The items were scored using a five-point Likert-type scale ranging from 1 “definitely does not apply” to 5 “definitely applies”. Cronbach’s alpha values for this scale were acceptable, ranging between 0.58 and 0.74. This is a reciprocal measure since it captures both parents’ efforts to promote a responsive and warm relationship and the child’s responsiveness to the parent. Higher scores reflect greater closeness.

### Covariates

***Child sex***. A binary variable was used to index the children’s biological sex, namely 1 “female” versus 0 “male”.

***Primary caregiver’s ethnicity***. A binary variable was utilised to record whether mothers identified as Irish (ethnic majority ‘*yes*’=1) versus ethnic minority (*‘yes’*=0).

***Household income at age 3***. Household income was coded using deciles, ranging from 1 “poorest” to 10 “richest”.

***Household type at age 3***. The household type was a binary variable capturing whether children lived in two-parent households (‘*yes’*=1) versus lone-parent households (‘*no’*=0).

***Child ill-health at age 3***. Children’s general ill-health was coded through an ordinal variable ranging from 1 “very healthy, no problems” to 4 “almost always unwell”.

***Parent general ill-health at age 3.*** Parents’ general ill-health was coded using an ordinal variable ranging from 1 “excellent” to 5 “poor”.

### Statistical analyses

Frequentist bivariate correlations and descriptive statistics were calculated to examine the associations between the key outcomes and the data distributions. Since the variables’ ranges are different from each other (see Table 1), we standardised each repeated measure across waves using the average sample standard deviation. This resulted in repeated measures with a total average variance of 1 across waves, but different variances within-wave [40], which allows the appropriate specification of Bayesian priors and facilitates convergence [41, 42].

Next, the STARTS model was specified and estimated using Bayesian structural equation modelling (BSEM) in the *blavaan 0.5-3* package [43] in the statistical language R [44]. The BSEM version of STARTS with four-wave panel data results in more stable parameter estimates [30, 40]. In the current specification of the STARTS model with four waves, several equality constraints were needed to achieve an empirically identified model. The following specifications were adopted. The completely stable traits (CST) for emotional symptoms and parent-child closeness were correlated [25]. Autoregressive traits at time *t* (ART_t_) were predicting subsequent ART_t+1_ through a VAR[1] autoregressive process and were equated over time. With two-year measurement intervals, the autoregressive function represents the two-year stability of the constructs, also known as the carryover effect [45].

Cross-lagged (spillover) effects were freely estimated and were placed on the occasion-specific ART_t_ factors, reflecting, thus, purely within-child and within-parent effects [28]. The correlations between state components (S_t_), which included measurement error, were estimated and equated across waves [19]. Autoregressive trait disturbances were correlated within-wave and equated cross-wave *t* ≥ 2 [28]. Non-linear constraints on the ART_t_ and disturbance parameters required in maximum likelihood estimation [19, 25] are not required in BSEM STARTS [30]. As a robustness check, the covariates were entered into the STARTS as standardised predictors of the observable variables to evaluate whether covariates changed the stability of the model. The regression coefficients of the covariates were held equal to reflect their time-invariant nature [45]. To evaluate whether the parameters were different from zero, we utilised the 95% Credible Interval (CrI).

The BSEM STARTS model was estimated using weakly informative priors to stabilise the estimation while not biasing the results [46]. These priors were drawn from past methodological evidence on the BSEM estimation of the STARTS model [30, 40] and Bayesian analysis in general. Specifically, the variances of the ART_t_, the CST, and the E_t_ were specified to follow an Inverse Gamma distribution with a prior sample size of *νφ* = 1 and variance of one-third [30, 40]. Given the absence of strong theoretical evidence, the regression coefficients for the cross-lagged and VAR[1] were specified to follow a generic weakly informative Normal prior ${\beta}_{t}\sim\;\text{N}(0, 1)$, which assumes that the coefficients are likely to range between − 1 and 1 [47]. The rest of the parameters were assumed to follow the default diffuse prior distributions in the software [43]. As a sensitivity analysis, the BSEM STARTS was also estimated using νφ = 3 and variance of one third, which is a good alternative [30, 40]. The results of the sensitivity analysis are presented in the Supplemental Materials.

To obtain Bayesian estimates from the posterior distribution, the Hamiltonian Markov Chain Monte Carlo (MCMC) method was deployed [48] in the *blavaan* package. Three MCMC chains were run with 1000 burnin samples and 1000 saved samples per chain. The convergence of the MCMC algorithm was evaluated using the Potential Scale Reduction (PSR) criterion, whereby values below 1.1 and preferably below 1.05 were taken to indicate convergence [49]. The efficiency of the posterior samples was examined using the Effective Sample Size (ESS), whereby ESS equal to/greater than 300 indicated the reliability of the posterior quantities [50].

Since the STARTS model is a latent variable model falling under the BSEM family, the Bayesian approximate and non-centrality fit indices were used to judge whether the model was a realistic enough representation of the data-generating processes [51]. Following Hu and Bentler (1999), we considered the following approximate indices as good fit: BCFI and BTLI values close to/ above 0.95 and BRMSEA values below 0.06. Values close to/above 0.95 in the non-centrality fit indices of BGammaHat and McDonald’s BMc were also considered indicators of very good fit [52]. Missing data were handled internally through full-information Bayesian estimation [53].

## Results

### Bivariate correlations and descriptive statistics

Frequentist bivariate correlations and descriptive statistics were calculated for all key outcomes and covariates (see Table 1). Additionally, the VIF coefficients were computed to evaluate the extent of potential multicollinearity. The average VIF was 1.44 (range 1.19–1.70), revealing no multicollinearity between the key indicators.

**Table 1:** Bivariate correlations and descriptive statistics for key outcomes and covariates

Variable	1	2	3	4	5	6	7	8	9	10	11	12	13	14	
1. Ethnic	1														
2. Income	0.125^***^	1													
3. Child sex	− 0.011	− 0.038^***^	1												
4. Household type	− 0.004	0.315^***^	− 0.033	1											
5. Child ill-health	− 0.008	− 0.029^*^	− 0.067^***^	− 0.069^***^	1										
6. Parent ill-health	− 0.042^***^	− 0.164^***^	0.031^*^	− 0.128^***^	0.191^***^	1									
7. EM1	0.006	− 0.095^***^	0.010	− 0.071^***^	0.186^***^	0.114^***^	1								
8. EM2	0.012	− 0.062^***^	0.008	− 0.112^***^	0.157^***^	0.124^***^	0.416^***^	1							
9. EM3	0.022	− 0.111^***^	0.005	− 0.094^***^	0.178^***^	0.136^***^	0.318^***^	0.446^***^	1						
10. EM4	0.043^***^	− 0.095^***^	0.012	− 0.131^***^	0.163^***^	0.146^***^	0.306^***^	0.445^***^	0.599^***^	1					
11. CLOSE1	0.011	0.034^*^	0.082^***^	0.023	− 0.090^***^	− 0.075^***^	− 0.121^***^	− 0.091^***^	− 0.096^***^	− 0.104^***^	1				
12. CLOSE2	0.016	0.019	0.096^***^	0.032	− 0.087^***^	− 0.094^***^	− 0.089^***^	− 0.172^***^	− 0.139^***^	− 0.147^***^	0.342^***^	1			
13. CLOSE3	0.092^***^	0.040^*^	0.121^***^	0.024	− 0.084^***^	− 0.127^***^	− 0.089^***^	− 0.140^***^	− 0.235^***^	− 0.166^***^	0.241^***^	0.380^***^	1		
14. CLOSE4	0.029	− 0.020	0.107^***^	0.049^***^	− 0.100^***^	− 0.083^***^	− 0.079^***^	− 0.128^***^	− 0.176^***^	− 0.198^***^	0.245^***^	0.369^***^	0.463^***^	1	
*Descriptive statistics*															
M (SD)	0.856 (0.35)	2.944 (1.41)	0.487 (0.50)	1.833 (0.37)	1.286 (0.51)	2.104 (0.94)	1.406 (1.42)	1.606 (1.73)	1.941 (2.06)	2.111 (2.08)	33.805 (1.91)	33.724 (1.99)	33.416 (2.36)	33.378 (2.35)	
Min-Max	0–1	1–10	0–1	1–2	1–4	1–5	0–10	0–10	0–10	0–10	17–35	15–35	9–35	7–35	

*n* = 7507; ***p <.001; **p <.01; *p <.05; M: Mean; SD: Standard deviation; Child sex: female vs. male; Ethnic: Ethnic majority (Irish) vs. ethnic minority; EMt: Child emotional difficulties at time t; CLOSEt: Parentchild closeness at time t

### Unconditional STARTS model of child emotional symptoms and parent-child closeness

The unconditional STARTS model estimation terminated normally. The maxPSR = 1.00 and the minESS = 709.582, indicated MCMC convergence and reliable posterior quantities, respectively. The model was re-fit using 3,000 samples drawn from each of the MCMC and the results remained unchanged. The trace plots also revealed a very good mixing of the chains supporting the convergence conclusion (see Supplemental Materials). Hence, convergence was confirmed. The STARTS model fitted the data very well according to the fit indices, BCFI = 1.00, BTLI = 1.00, BRMSEA = 0.009, BGammaHat = 0.999, McDonald’s BMc = 0.999. The posterior distribution histograms also indicated normally distributed estimates (see Supplemental Materials).

All parameter estimates were within the normal bounds and the standard deviations were reasonably small. The standardised latent variable loadings are presented in Table 2 to gauge the extent to which the constructs reflect more CST or ARTt variance. As shown in Table 2, emotional symptoms were characterised more by a predictable ART_t_. Closeness was more time-invariant between ages 3 years and 5 years, but was characterised more by ART_t_ between ages 7 years and 9 years.

**Table 2:** Standardised latent completely stable trait and autoregressive trait loadings

Path specification	Lambda	Path specification	Lambda
CST_EM ➔ EM1	0.600	ART_EM1 ➔ EM1	0.467
CST_EM ➔ EM2	0.494	ART_EM2 ➔ EM2	0.544
CST_EM ➔ EM3	0.410	ART_EM3 ➔ EM3	0.631
CST_EM ➔ EM4	0.404	ART_EM4 ➔ EM4	0.796
CST_CL ➔ CLOSE1	0.538	ART_CL1➔ CLOSE1	0.372
CST_CL ➔ CLOSE2	0.514	ART_CL2➔ CLOSE2	0.442
CST_CL ➔ CLOSE3	0.434	ART_CL3➔ CLOSE3	0.528
CST_CL ➔ CLOSE4	0.421	ART_CL4➔ CLOSE4	0.671

*N* = 7507; CST_EM: Completely stable trait emotional symptoms; CST_CL: Completely stable trait parent-child closeness ART_EM_t_: Autoregressive trait emotional symptoms at time t; ART_CL_t_: Autoregressive trait closeness at time t; Lambda: Factor loading

The unconditional STARTS parameter estimates of main interest are presented in Table 3. As shown in Table 3, the ART_t_s were strongly linked between occasions. In simple terms, this means that in the two-year interval between measurement waves, children’s emotional symptoms and parent-child closeness changed very slowly. The zero value was not included in 95% CrIs between ages 3 years and 7 years, indicating a bidirectional association over time between parent-child closeness and child emotional symptoms in childhood between ages 3 and 7. Having greater than normal parent-child closeness predicted less than usual child emotional difficulties. Similarly, greater than normal emotional symptoms predicted less than usual parent-child closeness.

An overall decline in the magnitude of the cross-lagged coefficients was also observed over time, which we describe as a ‘developmental decay’ in the reciprocal relations. The autoregressive trait disturbance correlations between ages 5 and 9 were all very weak, which signalled that there were few unaccounted influences on the ART_t_ VAR[1] process that might be shared. The latent states were rather weakly, and negatively correlated, illustrating that the completely time-specific components explained little shared variance. The CST correlation was moderate to large, indicating that parent-child relationships characterised by enduring closeness in childhood were associated with lower long-standing child emotional difficulties on average.

**Table 3:** Unconditional STARTS Model’s results

Path specification	Mean estimate (SD)	95% CrI for mean estimate		Standardised estimate
		LL	UL	
*Autoregressions*				
ART_EM1➔ ART_EM2	1.095 (0.056)	0.996	1.216	0.775
ART_EM2➔ ART_EM3	1.095 (0.056)	0.996	1.216	0.784
ART_EM3➔ ART_EM4	1.095 (0.056)	0.996	1.216	0.856
ART_CL1➔ ART_CL2	1.145 (0.082)	0.998	1.328	0.919
ART_CL2➔ ART_CL3	1.145 (0.082)	0.998	1.328	0.809
ART_CL3➔ ART_CL4	1.145 (0.082)	0.998	1.328	0.873
*Cross lagged regressions*				
ART_EM1➔ ART_CL2	− 0.585 (0.187)	-1.032	− 0.294	− 0.521
ART_EM2➔ ART_CL3	− 0.394 (0.070)	− 0.532	− 0.268	− 0.351
ART_EM3➔ ART_CL4	− 0.021 (0.040)	− 0.098	0.059	− 0.020
ART_CL1➔ ART_EM2	− 0.643 (0.192)	-1.089	-0.333	− 0.410
ART_CL2➔ ART_EM3	− 0.642 (0.135)	− 0.928	− 0.407	− 0.365
ART_CL3➔ ART_EM4	− 0.030 (0.050)	− 0.130	0.066	− 0.019
*Autoregressive traits correlation at age 3-years*				
ART_EM1— ART_CL1	0.113 (0.016)	0.084	0.148	0.940
*Autoregressive traits disturbance correlations*				
D_EM1— D_CL1	0.045 (0.011)	0.023	0.067	0.261
D_EM2— D_CL2	0.045 (0.011)	0.023	0.067	0.261
D_EM3— D_CL3	0.045 (0.011)	0.023	0.067	0.261
*State correlations*				
S_EM1— S_CL1	− 0.065 (0.006)	− 0.077	− 0.052	− 0.192
S_EM2— S_CL2	− 0.065 (0.006)	− 0.077	− 0.052	− 0.149
S_EM3— S_CL3	− 0.065 (0.006)	− 0.077	− 0.052	− 0.108
S_EM4— S_CL4	− 0.065 (0.006)	− 0.077	− 0.052	− 0.180
*Completely stable trait correlation*				
CST_EM— CST_CL	− 0.128 (0.016)	− 0.161	− 0.100	− 0.572

*N* = 7507; N Burnin = 1000; N Samples = 1000; Mean Estimate indicates average over the posterior; CrI = Credible Interval; *LL* = lower limit; *UL* = upper limit; ART_EMt: Autoregressive trait of emotional symptoms over time; ART_CLt: Autoregressive trait of parent-child closeness over time; D_EMt: Disturbance emotional symptoms; DCLt: Disturbance parent-child closeness; S_EMt: State emotional symptoms over time; S_CLt: State parent-child closeness over time; CST_EM: Stable trait emotional symptoms; CST_CL: Stable trait closeness; t = 1: age 3; t = 2: age 5; t = 3: age 7; t = 4: age 9; AR[1]: First-order autoregression; Arrows signify regressions; Hyphens indicate bivariate correlations

### Robustness check: conditioning the STARTS model on standard demographic covariates

To test whether the nature of the within-person relations between parent-child closeness and child emotional difficulties remained stable accounting for standard demographics, we ran a conditional STARTS model. The conditional STARTS achieved very good mixing and reliability with PSR = 1 and ESS = 713.103. The conditional model fitted the data very well, BRMSEA = 0.048, BCFI = 0.991, BTLI = 0.990, BMc = 0.902, and BGammaHat = 0.971. The results of the covariates’ effects on the observable variables in the conditional STARTS model are presented in Table 4 and the conditional STARTS parameters are presented in Table S1 in the Supplemental Materials. In short, we observed rather weak effects of most covariates, except for parent and child general ill-health that served as risk factors for increased emotional difficulties and decreased parent-child closeness. Female children were slightly at risk for increased emotional difficulties and benefited from increased parent-child closeness. Income and ethnicity had negligible effects.

**Table 4:** Covariate effects on the manifest variables as derived from the conditional STARTS model

Outcome variable	Predictor	Mean B (SD)	95% CrI	
			LL	UL
Emotional difficulties				
	Child sex (female)	0.027 (0.008)	0.012	0.042
	Ethnicity (ethnic majority)	0.014 (0.008)	− 0.001	0.029
	Household income age 3	− 0.046 (0.008)	− 0.063	− 0.030
	Household type	− 0.034 (0.008)	− 0.050	− 0.018
	Child ill-health	0.124 (0.008)	0.108	0.140
	Parent’s ill-health	0.069 (0.008)	0.053	0.085
Parent-child closeness				
	Child sex (female)	0.088 (0.008)	0.071	0.103
	Ethnicity (ethnic majority)	0.029 (0.008)	0.013	0.045
	Household income age 3	0.000 (0.009)	− 0.019	0.016
	Household type	0.013 (0.008)	− 0.003	0.029
	Child ill-health	− 0.058 (0.008)	− 0.074	− 0.042
	Parent’s health	− 0.064 (0.008)	− 0.080	− 0.048

*N* = 7507; N Burnin = 1000; N Samples = 1000; B Estimate indicates average over the posterior; SD: Standard deviation; CrI = Credible interval; *LL* = lower limit; *UL* = upper limit; Covariate variances were estimated with a diffuse conjugate prior σ^2^ ~ IG(0.01, 0.01) to avoid missing data due to covariates

Taken together, the unconditional and conditional models confirmed that the inclusion of the covariates did not change the conclusions drawn from the unconditional bivariate STARTS (Table 3) and the covariates had rather weak predictive effects.

## Discussion

Given the reported increases in children’s emotional difficulties [1], we drew upon large-scale data to examine the potential protective role of parent-child closeness in preventing child emotional difficulties and, concurrently, to estimate the influence of child emotional difficulties on the quality of parent-child relationships. Therefore, the study examined the bi-directional relations between parent-child closeness and child emotional symptoms using the bivariate STARTS model [19]. This modelling approach adds to the literature in multiple ways. First, the modelling verifies the connections between traits and states between pairs of time points, and between completely stable traits across all time points. Second, the study adds to the discussion on the existence of cross-lagged prospective effects between parent-child closeness and child emotional difficulties in the rather under-researched developmental period of early to middle childhood from a trait-state perspective. The findings are further unpacked and linked with previous literature below.

### Autoregressive effects over time

First, we found that children’s emotional difficulties and parent-child closeness across the developmental period of 3 years to 9 years (e.g., demonstrating strong autoregressive qualities) could also be partially characterised as predictable traits that slowly underwent change over time. In simple terms, the strong carryover effects suggest that increasing parent-child closeness and emotional difficulties in early childhood create persistent and strongly predictable changes later in childhood. The strength of the within-person carryover effects is unique since past empirical evidence has shown that both parent-child closeness and child emotional difficulties constitute rather weak to moderately stable over time [6, 9]. This finding of slow but predictable changes in childhood emotional difficulties can give us hope that, despite the genetic accounts of childhood mental health difficulties [54, 55], we can intervene and improve children’s emotional difficulties perhaps by investing in parent-child closeness and other positive development indicators. Next, we turn to the discussion of the longitudinal cross-lagged effects.

### Cross-lagged effects between parent-child closeness and child emotional difficulties

The study also showed that earlier emotional difficulties predicted later parent-child closeness, and vice versa, and that these cross-lagged associations were strongest from age 3 years to 5 years, weakening at age 5 years to 7 years, then disappearing at age 7 years to 9 years, demonstrating a weakening connection across time. Between these pairs of variables over time, we can conclude, based on the magnitude of the cross-lagged coefficients [27], that emotional difficulties appeared to be the dominant variable in this feedback loop that more strongly predicted decreases in parent-child closeness in early childhood and the transition to middle childhood. This suggests the need to further invest in the prevention of increasing emotional mental health symptoms as a key strategy for improving the quality of parent-child interactions.

Evidence on the bidirectional effects between parent-child closeness and child emotional difficulties has been rather inconclusive and mainly depends on the modelling choices of the researchers (between-person versus within-person approaches). The present findings are compatible with evidence provided by Chiang and Bai (2022), who reported a statistically significant reciprocal effect between closeness and depressive symptoms in adolescence. However, the present results contradict the null within-person effects between closeness and emotional difficulties, such as depressed affect or anxiety [8, 9]. Hence, our H1 was confirmed. At this stage, it should be mentioned that the current analyses are based on a larger than typical sample of children and families, which lends credibility to the results to a great extent.

It should be noted here that the discrepancies between the current findings and those arising from previous studies can possibly be attributed to different modelling choices. Specifically, in the current study, we utilised the STARTS model, which separates measurement error [56] and is more robust to regression to the mean effects [57], whereas previous research did not account for measurement error [7–9, 18]. Additionally, the discrepancies regarding the cross-lagged effects might be connected with measurement decisions; that is, the different findings might be related to the different measures utilised or the representativeness of the samples.

This ‘developmental decay’ effect suggests that the years between age 3 and age 7 are crucial for children’s emotional difficulties and parent-child relationships. Between years 3 and 7, we observed that stronger than usual parent-child closeness negatively affected subsequent within-child emotional difficulties, which confirms our H2 and rejects H3. This finding is compatible with developmental theory, which suggests that the parent-child relationships during the early years are more important for later-life adjustment [58]. An alternative explanation for this ‘developmental decay’ effect might be linked with the time parents spend with their children; that is, parents tend to spend more shared time with younger children [59] and our hypothesis here is that this shift in shared time in middle childhood might underpin the disappearance of the structural relations between child emotional difficulties and parent-child closeness. The lack of a bidirectional association between ages 7 and 9 is particularly interesting because the transactional model of child psychopathology development suggests the omnipresent existence of bidirectional associations between children and their families [5]. This might indicate that researchers need to delve deeper into these associations to gain a greater understanding of the factors that might disrupt these effects over time.

In addition to the above, the STARTS model revealed that greater-than-usual emotional difficulties predicted less than usual parent-child closeness, which confirmed H4 and rejected H5. This finding is compatible with related past empirical studies [6, 16] that showed a negative predictive effect from child emotional difficulties to parent-child closeness. On one hand, a potential explanation for this finding is that this inverse relation occurred due to the reciprocal nature of the parent-child closeness scale. On the other hand, higher than normal internalising symptoms (a broad umbrella of emotional and mood symptoms) have not been associated with greater than usual prosociality in children and adolescents [4]. Practically, this means that children with heightened emotional difficulties might be typically more withdrawn from the parent-child relationship and might not seek the comfort of closeness. Psychopathology research with adults has also illustrated that adults, who exhibit greater depression scores, were also characterised by greater introversion [60]. Similar evidence comes from meta-analytic work suggesting that emotional difficulties were positively linked with insecure attachment to the parent/ caregiver, characterised by less willingness to form close relationships [61]. Hence, H4 holds greater merit compared to H5.

At this point, we ought to note that the current study investigated the longitudinal relations between parent-child closeness and child emotional difficulties in the crucial formative years from early to middle childhood, whereas previous empirical work has employed within-child and within-parent designs targeting adolescents [6, 8, 9]. This suggests that the current study offers new insights beyond what is previously available in the literature on these relations from a trait-state perspective.

### Completely stable traits and contemporaneous state correlations between parent-child closeness and child emotional difficulties

In contrast to past studies, we also examined the structural relations between parent-child closeness at the CST and the State levels. It should be underscored that the current findings present a more holistic picture of the long-term association between parent-child closeness and child emotional difficulties due to the use of the Bayesian STARTS model [30]. The STARTS model offers the advantage of a clear separation of CST, ARTs, and States (the latter including random and measurement error), which allows the estimation of the association between closeness and child emotional difficulties at different time units [19]. This also contributes to the existing literature examining this topic. In short, the analyses showed that the correlation between the emotional difficulties and the closeness CSTs was negative and of moderate strength, whereas the association between the State emotional difficulties and State closeness was negative and rather weak over time. These truly unique findings provide strong evidence in favour of a negative association overall.

A similar negative association between CSTs, though via a different analytic model, has been reported in past research [9]. The first result suggests that developing stable high emotional difficulties is associated on average with less parent-child closeness from early to middle childhood. This finding indicates that the association between closeness and emotional mental health is rather weak cross-sectionally at specific ages. Hence, interventions targeting parent-child relationships and child emotional mental health should aim for long-term positive outcomes, rather than short-term benefits.

### Strengths, limitations, and future directions

The current study has several methodological and conceptual strengths. For instance, the large sample size, the robust and well-validated measures, and the longitudinal cohort design are among the unquestionable strengths of the current investigation. However, as with all empirical studies, some limitations ought to be noted. First, the current study utilised secondary data that included a specific number of available waves and instruments. By using more time-intensive data, future research will be able to shed more detailed insights into the momentary dynamics between parent-child closeness and child emotional difficulties.

Second, the parent-child closeness measure is a rather reciprocal measure of the quality of parent-child warm relationships and we recommend a replication of the findings with other measures, as well. Third, we suggest further research to replicate the current findings with other psychometric tools designed to measure child emotional symptoms, such as the Child Behavior Checklist [62]. Fourth, it is also important to study how these relations might change or remain stable when child emotional difficulties are teacher-reported since the SDQ has also a teacher/ carer version [63]. Finally, given the short-term four-wave longitudinal design, it is important to cross-validate the findings with longer repeated measures and across different developmental periods (e.g., adolescence).

### Implications

Given the overall significance of the present results, some implications might arise for both families and mental health practitioners. Specifically, investing more qualitative shared time with children that is characterised by warmth and closeness might prove helpful in reducing children’s emotional mental difficulties over time. Additionally, given the decay in the reciprocal relations between ages 7 and 9 years old, we recommend more interventions that would focus on strengthening the parent-child closeness before the stage of middle childhood when the relations between closeness and child emotional difficulties are more robust. It cannot be stressed enough how important positive parent-child interactions are for reducing child emotional difficulties [64]. Hence, effective family-based systemic interventions can be used to improve the quality of parent-child relationships and reduce potential negative consequences of child emotional difficulties [65]. However, child psychological resilience occurs as the outcome of multiple interactions between different systems and agents [32]. Therefore, teachers could also play a role in the timely identification of children struggling with emotional difficulties, which could potentially help improve early detection, intervention, and subsequent, improvement of parent-child closeness.

## Conclusion

Childhood is a pivotal time for an individual’s emotional and social development. In this study, we illustrated that in early childhood (ages 3 years up to 7 years), children’s emotional difficulties and parent-child closeness have the strongest impact on each other, compared to middle childhood (ages 7 years up to 9 years). This result signals a need for interventions aimed at parents of 3–7-year-olds, that are designed to help parents minimise the negative impact of their children’s emotional difficulties on the emotional support they give to their child. These interventions will need to achieve long-term outcomes of improved parent-child relationships and reduced child emotional difficulties.

### Electronic supplementary material

Below is the link to the electronic supplementary material.

Supplementary Material 1

## Data Availability

The anonymised micro-data file was obtained from the Irish Social Sciences Data Archive (ISSDA) which contains composite variables only (https://www.ucd.ie/issda/data/guiinfant/). All eligible researchers can request access through the ISSDA.
