# Supplementary material for: Longitudinal relations between child emotional difficulties and parent-child closeness: a stability and malleability analysis using the STARTS model
Source: Child Adolesc Psychiatry Ment Health. 2024 Jul 15;18:85. doi: 10.1186/s13034-024-00777-1 (PMC11251125; doi:10.1186/s13034-024-00777-1)
Supplement: Supplementary file 1 — Supplementary Material 1 [file 13034_2024_777_MOESM1_ESM.docx]

Bayesian STARTS model of child emotional symptoms and parent-child closeness

PART A: Item wordings for the key scales

PART B: Bayesian diagnostic plots

PART C: Conditional STARTS model’s key parameters’ results

PART D: Sensitivity analysis of unconditional STARTS with prior sample size of νφ=3 and variance one third

PART A: Item wordings for key psychometric scales

Parent-child closeness (Pianta Scale)

| I share an affectionate relationship  [the Child] will seek comfort from me if upset  [the Child] values his/her relationship with me  [the Child] praise, beams with pride  [the Child] spontaneously shares information  It is easy to tune in to child’s feelings  [the Child] openly shares feelings and experiences |
| --- |

Emotional difficulties scale (Strengths and Difficulties Scale)

| [the Child] Often complains of headaches  [the Child] has many worries  [the Child] is often unhappy, downhearted  [the Child] is nervous or clingy in new situations  [the Child[ has many fears, easily scared |
| --- |

PART B:

Bayesian trace plots for key bivariate STARTS parameter estimates


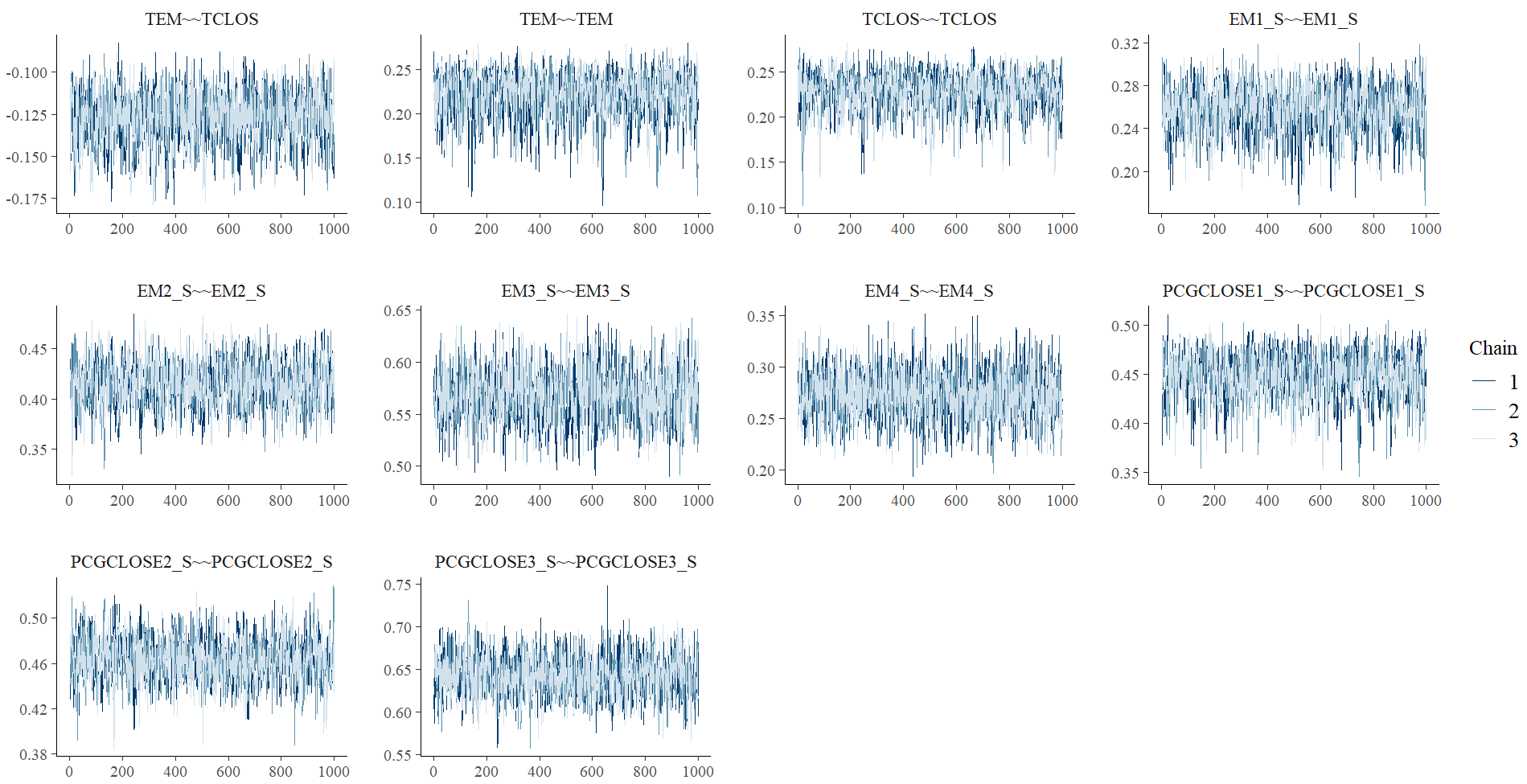


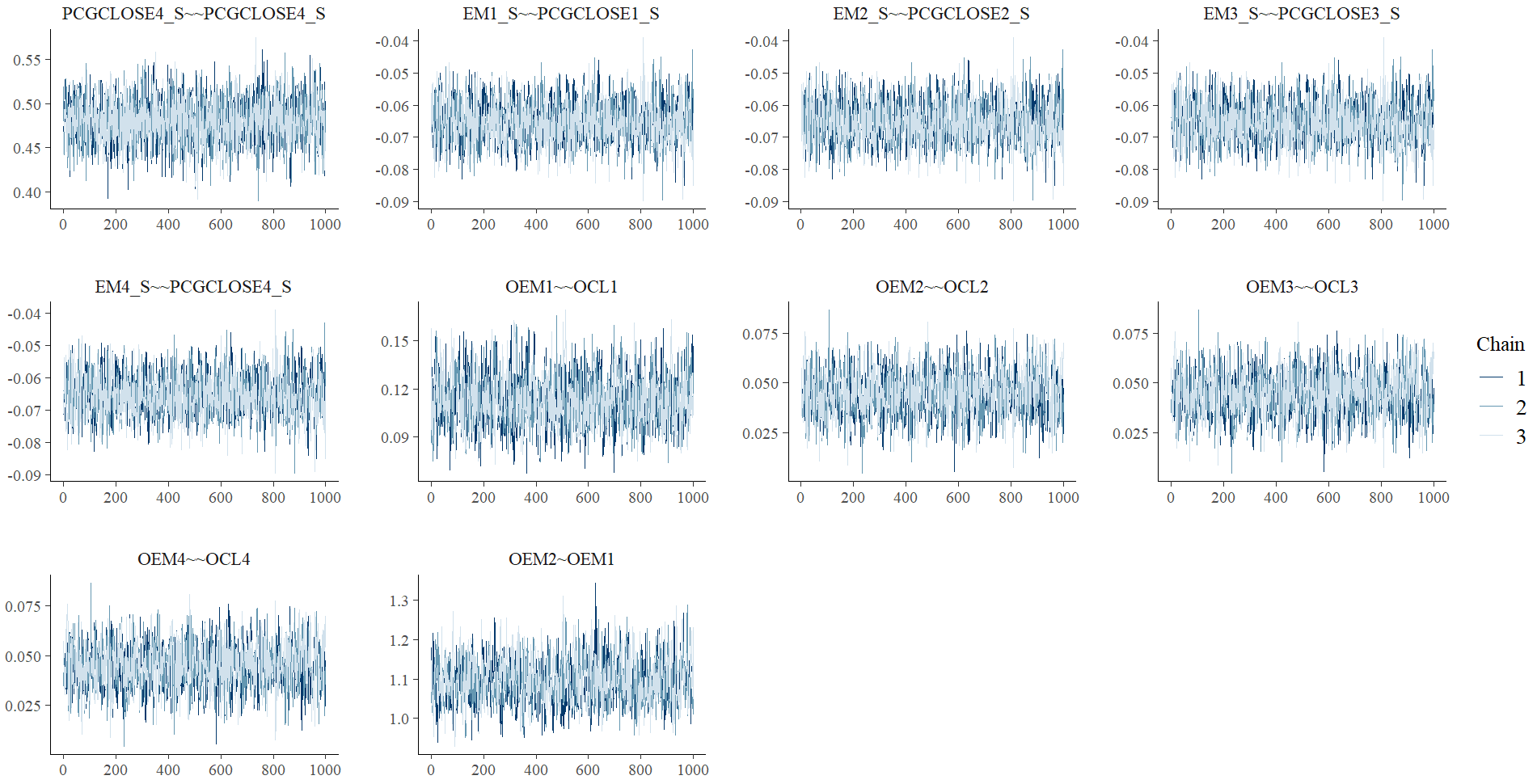


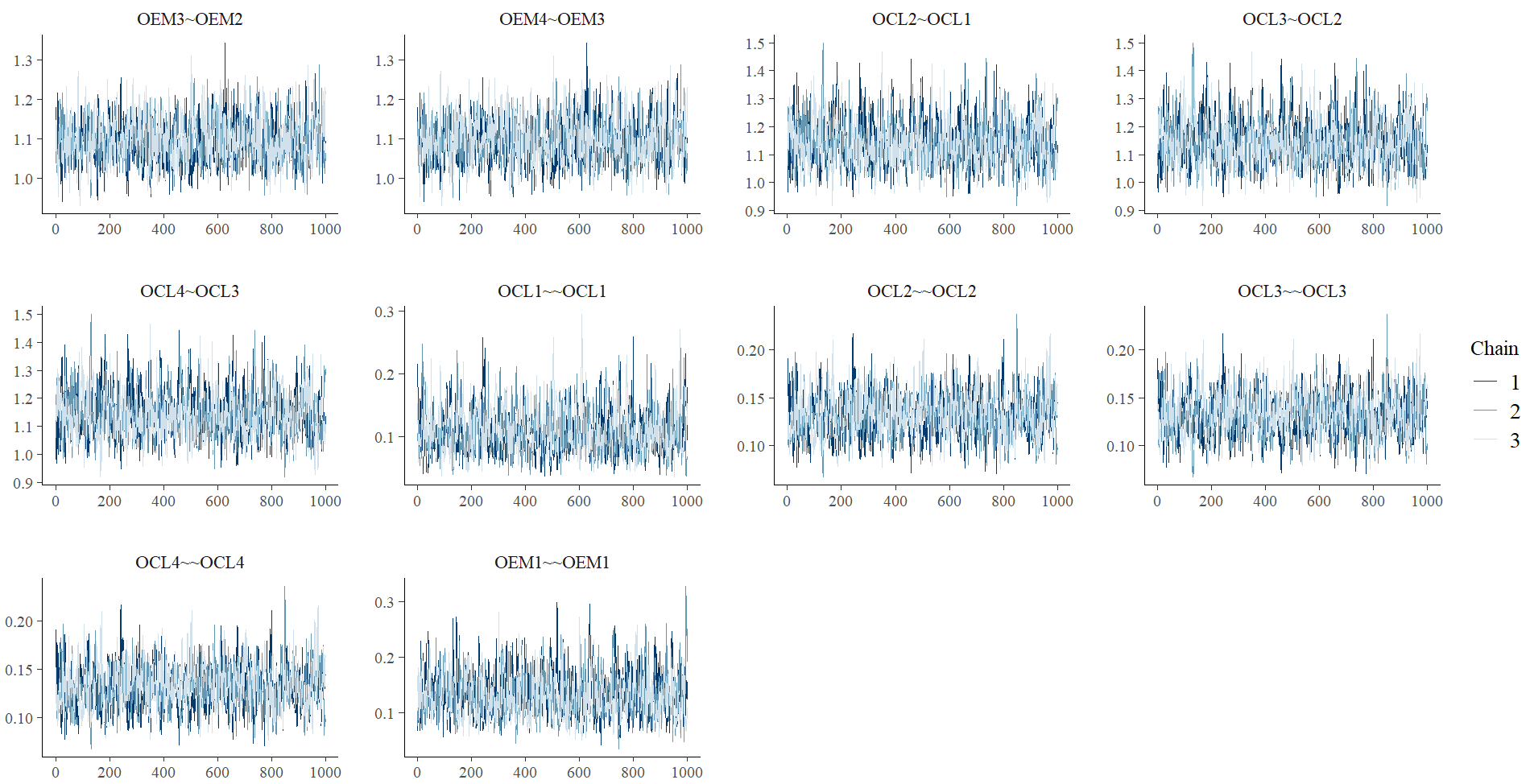


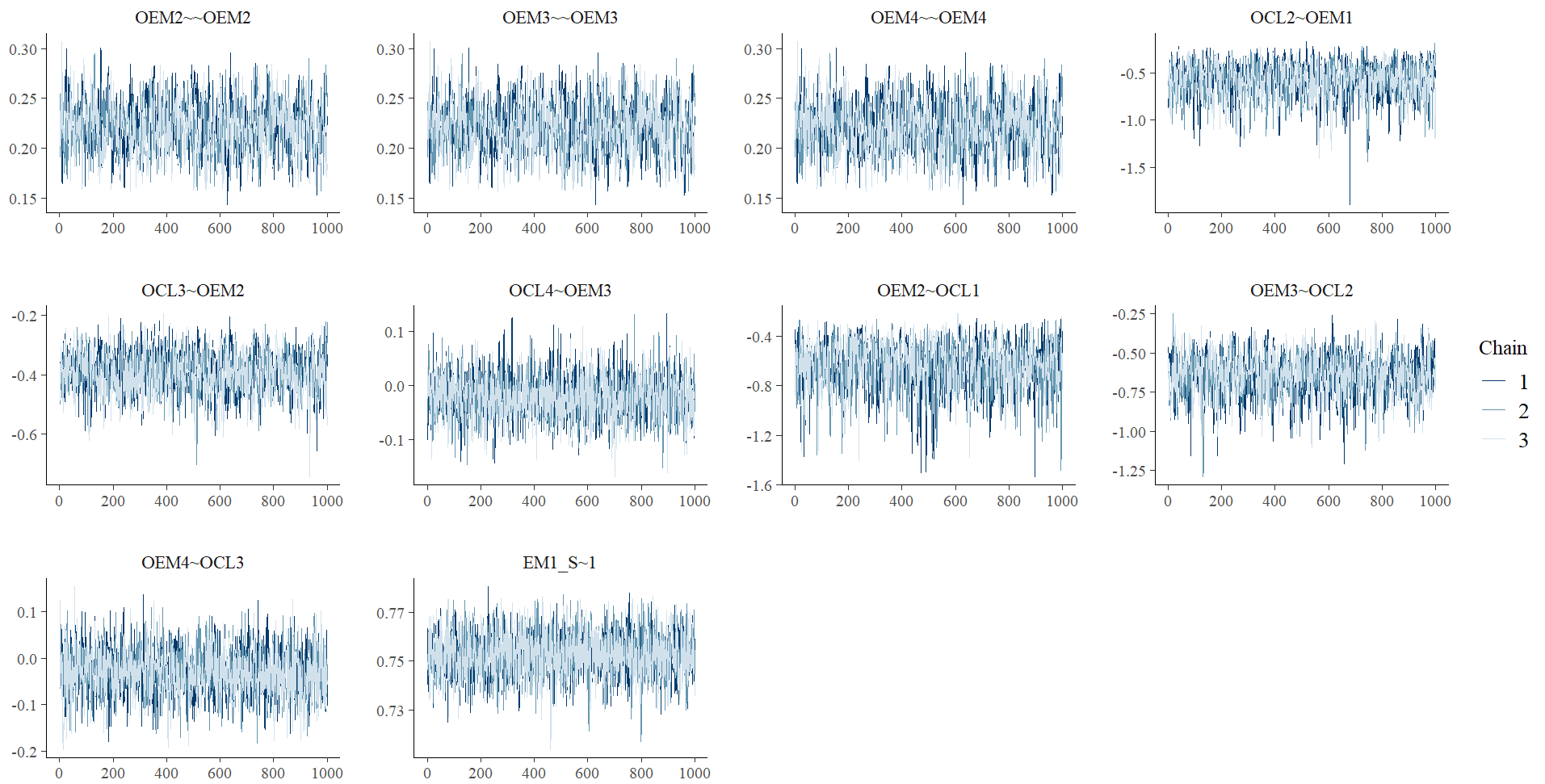


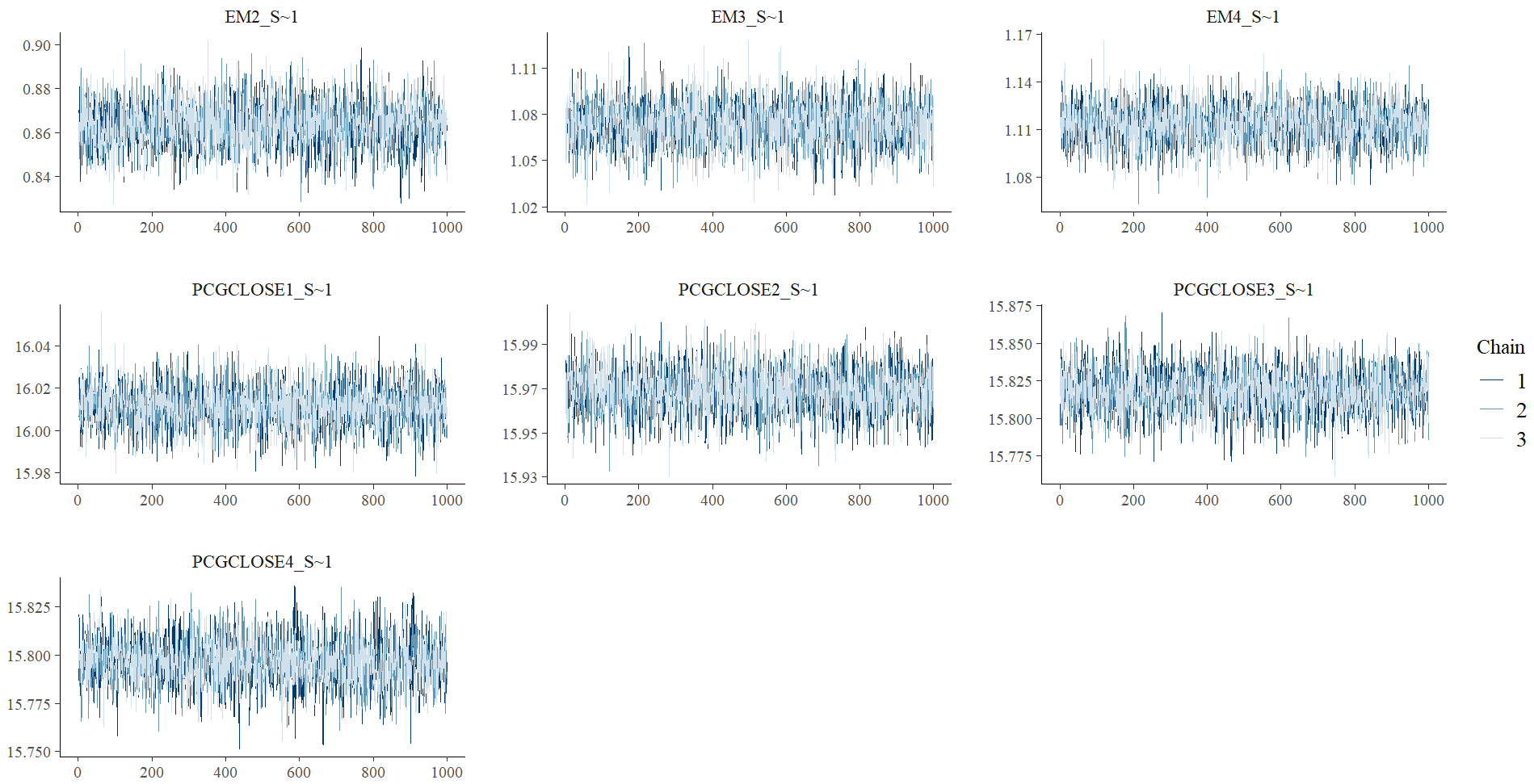


PART B: Bayesian posterior distribution histograms per parameter estimate


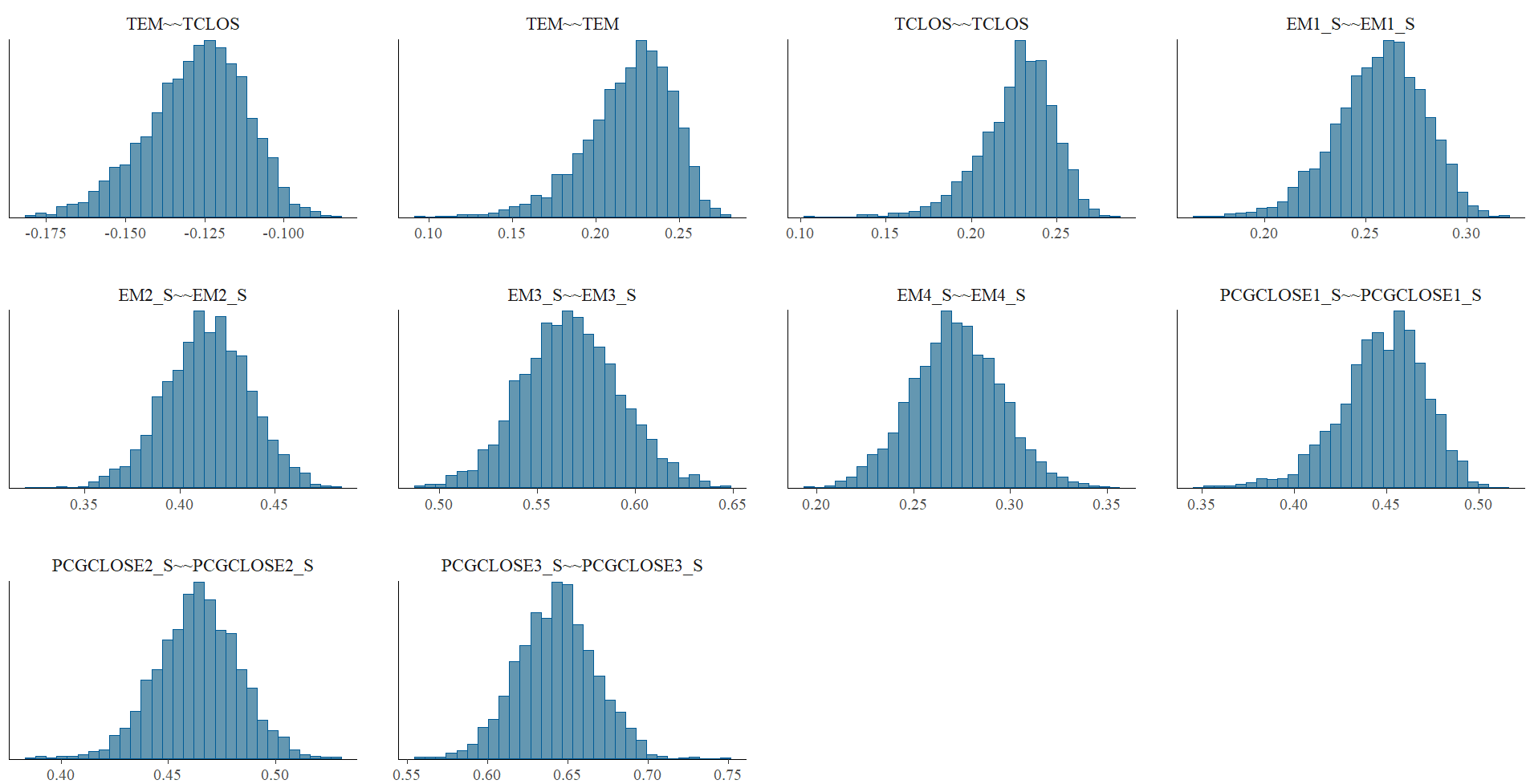


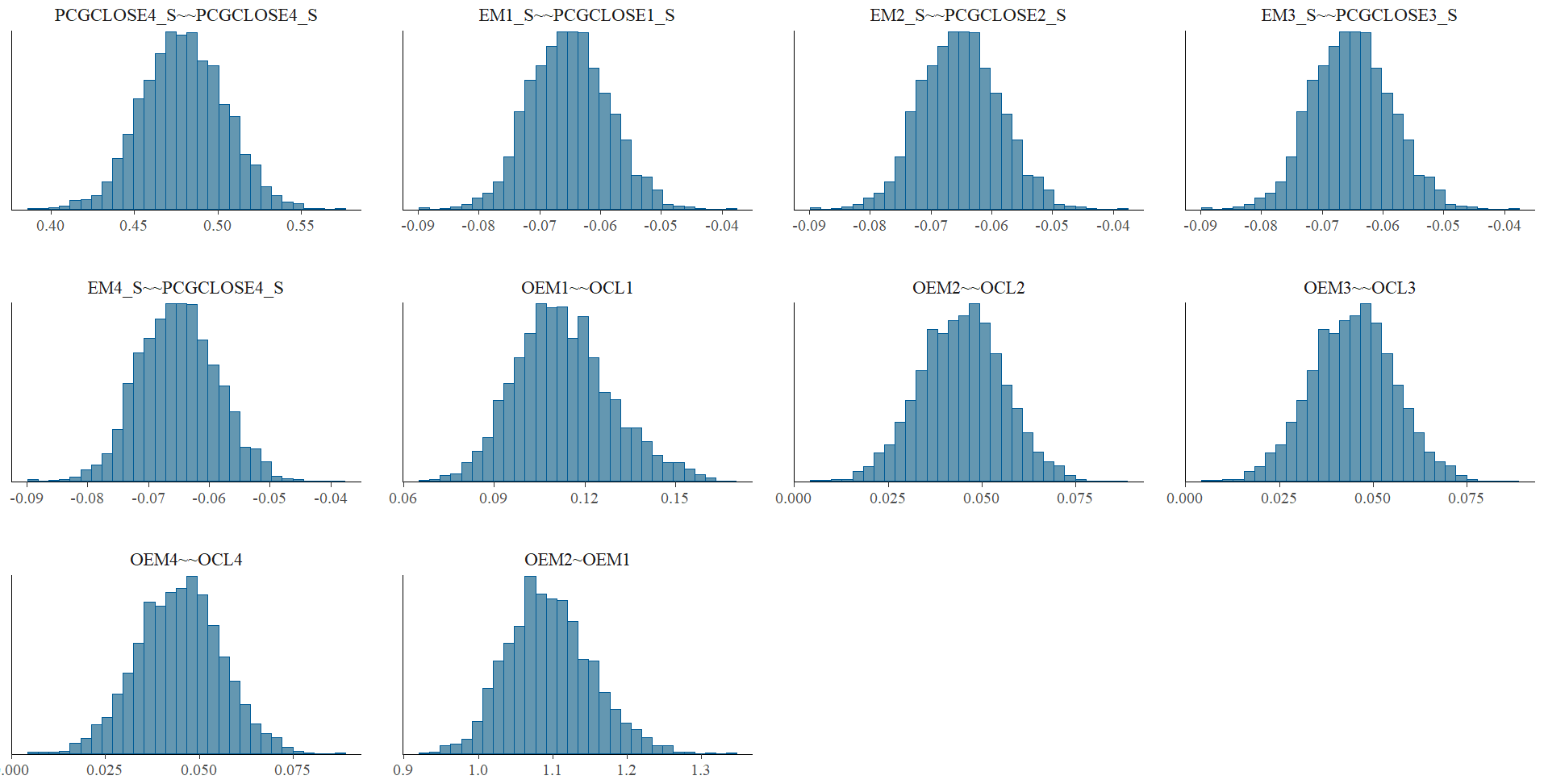


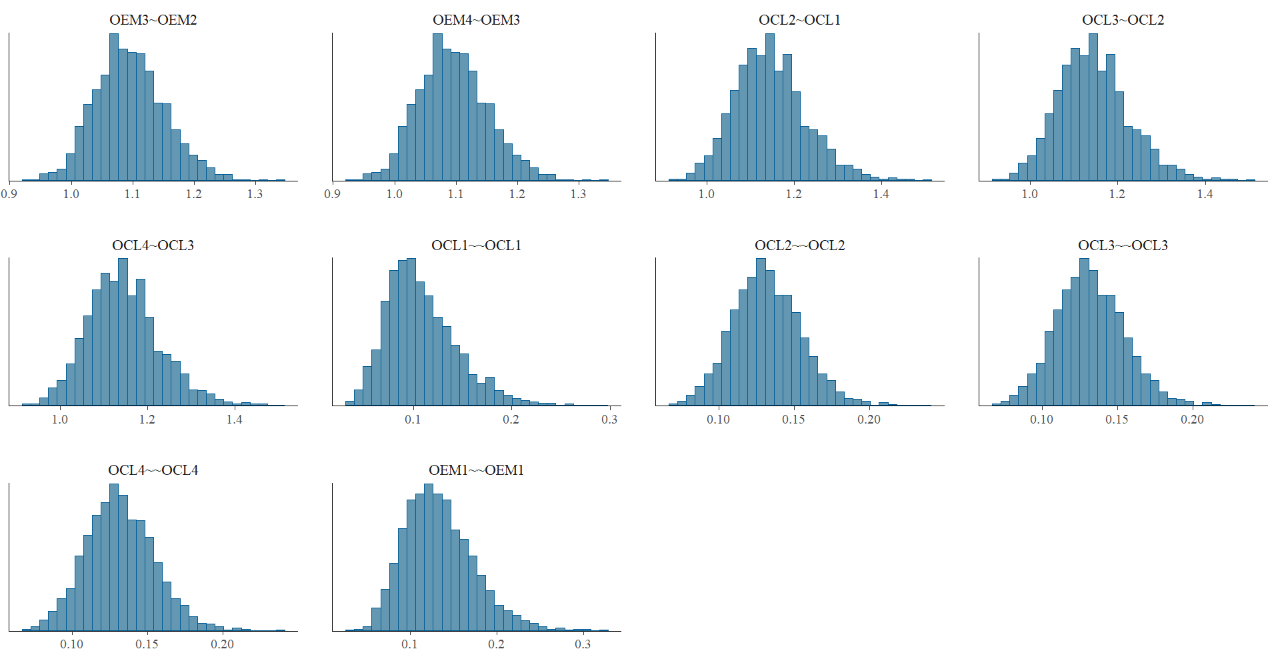


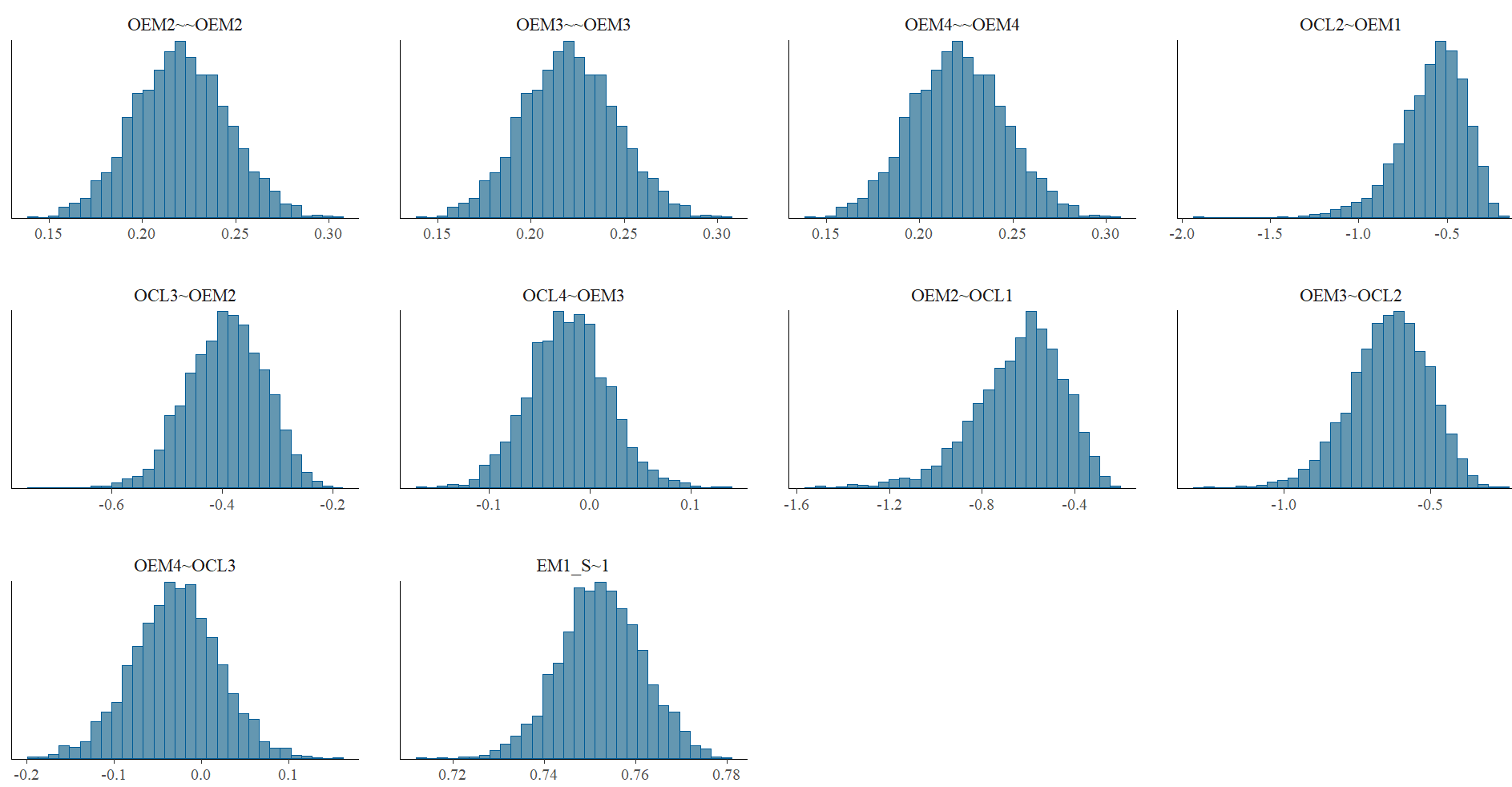


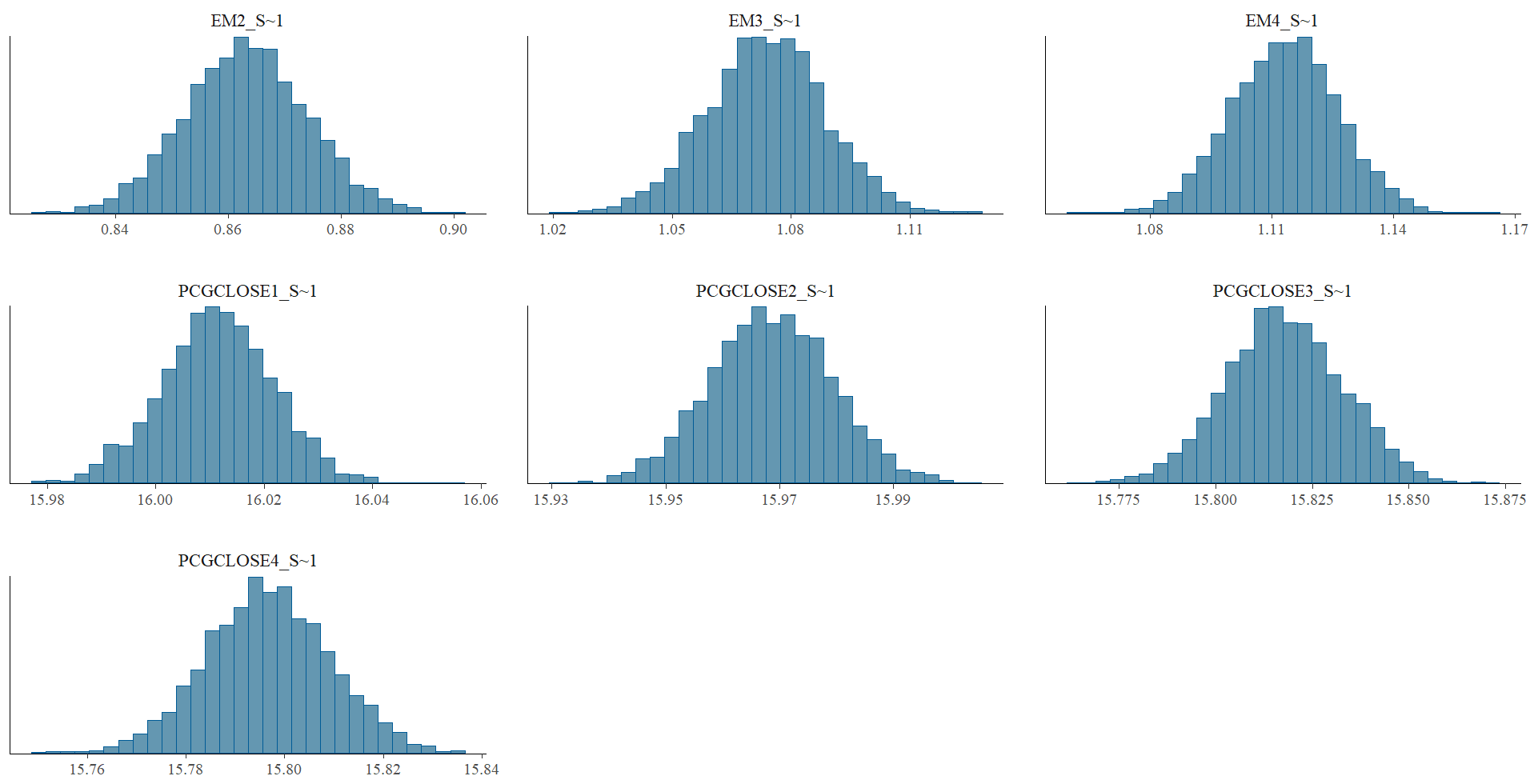


PART C: CONDITIONAL STARTS’ PARAMETERS

Table S1. Conditional STARTS model’s parameter estimates

|  | Estimate | | Post.SD | | 95% CrI | | | | β | |
| --- | --- | --- | --- | --- | --- | --- | --- | --- | --- | --- |
|  |  | |  | | LL | | UL | |  | |
|  | ART | | | | | | | | | |
| ART_EM2 ~ |  |  | |  | |  | |  | |  |
| ART_EM1 | 1.084 | 0.057 | | 0.985 | | 1.203 | | 0.807 | |  |
| ART_EM3 ~ |  |  | |  | |  | |  | |  |
| ART_EM2 | 1.084 | 0.057 | | 0.985 | | 1.203 | | 0.796 | |  |
| ART_EM4 ~ |  |  | |  | |  | |  | |  |
| ART_EM3 | 1.084 | 0.057 | | 0.985 | | 1.203 | | 0.849 | |  |
| ART_CL2 ~ |  |  | |  | |  | |  | |  |
| ART_CL1 | 1.185 | 0.088 | | 1.035 | | 1.371 | | 0.945 | |  |
| ART_CL3 ~ |  |  | |  | |  | |  | |  |
| ART_CL2 | 1.185 | 0.088 | | 1.035 | | 1.371 | | 0.834 | |  |
| ART_CL4 ~ |  |  | |  | |  | |  | |  |
| ART_CL3 | 1.185 | 0.088 | | 1.035 | | 1.371 | | 0.881 | |  |
| Cross-lagged | | | | | | | | | |  |
| ART_CL2 ~ |  |  | |  | |  | |  | |  |
| ART_EM1 | -0.53 | 0.162 | | -0.913 | | -0.282 | | -0.531 | |  |
| ART_CL3 ~ |  |  | |  | |  | |  | |  |
| ART_EM2 | -0.396 | 0.069 | | -0.535 | | -0.27 | | -0.376 | |  |
| ART_CL4 ~ |  |  | |  | |  | |  | |  |
| ART_EM3 | -0.031 | 0.04 | | -0.111 | | 0.052 | | -0.03 | |  |
| ART_EM2 ~ |  |  | |  | |  | |  | |  |
| ART_CL1 | -0.69 | 0.203 | | -1.162 | | -0.378 | | -0.408 | |  |
| ART_EM3 ~ |  |  | |  | |  | |  | |  |
| ART_CL2 | -0.698 | 0.139 | | -0.994 | | -0.458 | | -0.38 | |  |
| ART_EM4 ~ |  |  | |  | |  | |  | |  |
| ART_CL3 | -0.048 | 0.052 | | -0.149 | | 0.054 | | -0.029 | |  |
|  | Stable Trait Correlations | | | | | | | | | |
| CST_EM ~~ |  | |  | |  | |  | |  | |
| CST_CL | -0.118 | | 0.015 | | -0.15 | | -0.091 | | -0.602 | |
|  | State Correlations | | | | | | | | | |
| S_EM1 ~~ |  | |  | |  | |  | |  | |
| S_CL1 | -0.068 | | 0.006 | | -0.08 | | -0.055 | | -0.198 | |
| S_EM2 ~~ |  | |  | |  | |  | |  | |
| S_CL2 | -0.068 | | 0.006 | | -0.08 | | -0.055 | | -0.153 | |
| S_EM3 ~~ |  | |  | |  | |  | |  | |
| S_CL3 | -0.068 | | 0.006 | | -0.08 | | -0.055 | | -0.112 | |
| S_EM4 ~~ |  | |  | |  | |  | |  | |
| S_CL4 | -0.068 | | 0.006 | | -0.08 | | -0.055 | | -0.187 | |
|  | ART (disturbance) correlations | | | | | | | | | |
| ART_EM1 ~~ |  | |  | |  | |  | |  | |
| ART_CL1 | 0.117 | | 0.016 | | 0.089 | | 0.15 | | 0.95 | |
| D_EM2 ~~ |  | |  | |  | |  | |  | |
| D_CL2 | 0.049 | | 0.011 | | 0.028 | | 0.07 | | 0.301 | |
| D_EM3 ~~ |  | |  | |  | |  | |  | |
| D_CL3 | 0.049 | | 0.011 | | 0.028 | | 0.07 | | 0.301 | |
| D_EM4 ~~ |  | |  | |  | |  | |  | |
| D_CL4 | 0.049 | | 0.011 | | 0.028 | | 0.07 | | 0.301 | |

*Note*: N = 7507; N Burnin = 1000; N Samples = 1000; B Estimate indicates average over the posterior; CrI = Credible Interval; *LL* = lower limit; *UL* = upper limit; ART_EMt: Autoregressive trait of emotional symptoms over time; ART_CLt: Autoregressive trait of parent-child closeness over time; D_EMt: Disturbance emotional symptoms; DCLt: Disturbance parent-child closeness; S_EMt: State emotional symptoms over time; S_CLt: State parent-child closeness over time; CST_EM: Stable trait emotional symptoms; CST_CL: Stable trait closeness; t = 1: age 3; t = 2: age 5; t = 3: age 7; t = 4: age 9; AR[1]: First-order autoregression; ~ signify regressions; ~~ indicate bivariate correlations.

PART D: UNCONDITIONAL STARTS MODEL WITH PRIOR SAMPLE SIZE (νφ=3) PRIOR FOR VARIANCES

|  | Estimate | Post.SD | 95% CrI | | β |
| --- | --- | --- | --- | --- | --- |
|  |  |  | LL | UL |  |
|  | ART | | | | |
| ART_EM2 ~ |  |  |  |  |  |
| ART_EM1 | 1.092 | 0.061 | 0.986 | 1.23 | 0.783 |
| ART_EM3 ~ |  |  |  |  |  |
| ART_EM2 | 1.092 | 0.061 | 0.986 | 1.23 | 0.777 |
| ART_EM4 ~ |  |  |  |  |  |
| ART_EM3 | 1.092 | 0.061 | 0.986 | 1.23 | 0.858 |
| ART_CL2 ~ |  |  |  |  |  |
| ART_CL1 | 1.176 | 0.096 | 1.01 | 1.39 | 0.919 |
| ART_CL3 ~ |  |  |  |  |  |
| ART_CL2 | 1.176 | 0.096 | 1.01 | 1.39 | 0.812 |
| ART_CL4 ~ |  |  |  |  |  |
| ART_CL3 | 1.176 | 0.096 | 1.01 | 1.39 | 0.885 |
|  | Cross-lagged effects (ART) | | | | |
| ART_CL2 ~ |  |  |  |  |  |
| ART_EM1 | -0.576 | 0.2 | -1.082 | -0.274 | -0.542 |
| ART_CL3 ~ |  |  |  |  |  |
| ART_EM2 | -0.395 | 0.071 | -0.545 | -0.264 | -0.358 |
| ART_CL4 ~ |  |  |  |  |  |
| ART_EM3 | -0.012 | 0.042 | -0.087 | 0.074 | -0.012 |
| ART_EM2 ~ |  |  |  |  |  |
| ART_CL1 | -0.715 | 0.231 | -1.243 | -0.334 | -0.426 |
| ART_EM3 ~ |  |  |  |  |  |
| ART_CL2 | -0.692 | 0.152 | -1.027 | -0.423 | -0.375 |
| ART_EM4 ~ |  |  |  |  |  |
| ART_CL3 | -0.024 | 0.053 | -0.127 | 0.083 | -0.015 |
|  | Stable Trait Correlations | | | | |
| CST_EM ~~ |  |  |  |  |  |
| CST_CL | -0.124 | 0.015 | -0.156 | -0.097 | -0.549 |
|  | State correlations | | | | |
| S_EM1 ~~ |  |  |  |  |  |
| S_CL1 | -0.064 | 0.006 | -0.076 | -0.052 | -0.189 |
| S_EM2 ~~ |  |  |  |  |  |
| S_CL2 | -0.064 | 0.006 | -0.076 | -0.052 | -0.145 |
| S_EM3 ~~ |  |  |  |  |  |
| S_CL3 | -0.064 | 0.006 | -0.076 | -0.052 | -0.106 |
| S_EM4 ~~ |  |  |  |  |  |
| S_CL4 | -0.064 | 0.006 | -0.076 | -0.052 | -0.178 |
|  | ART (disturbance) correlations | | | | |
| ART_EM1 ~~ |  |  |  |  |  |
| ART_CL1 | 0.107 | 0.016 | 0.078 | 0.14 | 0.928 |
| D_EM2 ~~ |  |  |  |  |  |
| D_CL2 | 0.044 | 0.01 | 0.023 | 0.064 | 0.263 |
| D_EM3 ~~ |  |  |  |  |  |
| D_CL3 | 0.044 | 0.01 | 0.023 | 0.064 | 0.263 |
| D_EM4 ~~ |  |  |  |  |  |
| D_CL4 | 0.044 | 0.01 | 0.023 | 0.064 | 0.263 |

*Note*: N = 7507; N Burnin = 1000; N Samples = 1000; B Estimate indicates average over the posterior; CrI = Credible Interval; *LL* = lower limit; *UL* = upper limit; ART_EMt: Autoregressive trait of emotional symptoms over time; ART_CLt: Autoregressive trait of parent-child closeness over time; D_EMt: Disturbance emotional symptoms; DCLt: Disturbance parent-child closeness; S_EMt: State emotional symptoms over time; S_CLt: State parent-child closeness over time; CST_EM: Stable trait emotional symptoms; CST_CL: Stable trait closeness; t = 1: age 3; t = 2: age 5; t = 3: age 7; t = 4: age 9; AR[1]: First-order autoregression; ~ signify regressions; ~~ indicate bivariate correlations.
